# Oral Health Effects Among Adults Switching from Cigarettes to on!® Nicotine Pouches Compared to Those Who Continue Smoking

**DOI:** 10.3290/j.ohpd.c_1925

**Published:** 2025-03-25

**Authors:** Jianmin Liu, Jeffery S. Edmiston, Jingzhu Wang, Kimberly R. Milleman, Jeffery L. Milleman, Abigale L. Yoder, Maria Gogova, Mohamadi A. Sarkar

**Affiliations:** a Jianmin Liu Researcher, Study Leader, Altria Client Services LLC., Richmond, VA, USA. Conceptualisation, supervised conducting the study and statistical analysis, interpreted results, wrote the manuscript.; b Jeffery S. Edmiston Researcher, Altria Client Services LLC., Richmond, VA, USA. Conceptualisation, provided input on statistical analysis and interpretation of results, wrote the manuscript.; c Jingzhu Wang Statistician, Altria Client Services LLC., Richmond, VA, USA. Supervised the development of statistical analysis plan, contributed to the analysis and interpretation of the findings.; d Kimberly R. Milleman Dental Hygienist, Dental Site Lead, Primary Examiner, Salus Research, Inc., Fort Wayne, IN, USA. Conceptualisation, supervised conducting the study, statistical analysis, and interpreted results.; e Jeffery L. Milleman Dentist, Dental Site Lead, Principal Investigator, Salus Research, Inc., Fort Wayne, IN, USA. Conceptualisation, supervised conducting the study, statistical analysis, and interpreted results.; f Abigale L. Yoder Clinical Coordinator, Salus Research, Inc., Fort Wayne, IN, USA. Assisted in writing the clinical study report and manuscript.; g Maria Gogova Researcher, Altria Client Services LLC., Richmond, VA, USA. Conceptualisation, wrote the manuscript.; h Mohamadi A. Sarkar Fellow and Researcher, Regulatory Affairs, Altria Client Services LLC; Affiliate Professor of Clinical Pharmacology, Virginia Commonwealth University, Richmond, VA, USA. Conceptualisation, wrote the manuscript.

**Keywords:** cigarettes, gingivitis, harm reduction, nicotine pouches, oral health, oral tobacco-derived nicotine.

## Abstract

**Purpose:**

The oral-health impact of nicotine pouches, an emerging category of oral tobacco products, has not been well studied. We evaluated the effects of switching from cigarettes to on!® nicotine pouches (test product, TPs) on oral-health endpoints among adult smokers (AS) relative to those who just continued smoking (CS).

**Materials and Methods:**

In this randomised, open-label, parallel-group study, participants were randomly assigned to ad libitum use of 2, 4, or 8 mg nicotine TP or CS for 24 weeks. Oral-health endpoints, e.g., Modified Gingival Index (MGI), Gingival Bleeding Index (BI), and Lobene Stain Index (LSI)] were assessed at baseline and weeks 12 and 24, and compared between the TP and CS groups by using linear mixed model analysis for repeated measurements.

**Results:**

n = 155 participants were randomised; 100 (TP = 48; CS = 52) completed week 12, and 85 (TP = 40; CS = 45) completed week 24 assessments. The TP group reduced their cigarette consumption by >90% by weeks 12 and 24 despite not intending to quit at baseline. Statistically significant reductions (p  <  0.001) were observed for MGI and BI at weeks 12 (MGI = 20%; BI = 30%) and 24 (MGI = 28%; BI = 23%) in the TP group compared to the CS group, as well as compared to baseline. Statistically significant reductions (~60%, p < 0.001) were also observed for LSI in TP vs CS. No statistically significant changes were observed for LSI in the CS group at weeks 12 and 24 compared to baseline.

**Discussion:**

The findings from this study suggest that TPs do not negatively impact users’ oral health over 24 weeks of use. The reduction in oral health endpoints supports the harm reduction potential of TPs.

Combustible cigarettes are the most harmful tobacco products, resulting in serious diseases like lung cancer, chronic obstructive pulmonary disease (COPD), and cardiovascular diseases. The smoking-related morbidity and mortality is caused by inhaling the smoke which consists of thousands of toxic chemicals. Adults who smoke (AS) are exposed to thousands of chemicals present in the smoke, many of which are identified as carcinogenic.^
39
^ The oral cavity is the first site of exposure to these chemicals which can negatively impact oral health, of which oral cancer is the most significant outcome.

Previous studies have shown a consistent association between cigarette use and elevated risks of oral cancer.^
2,4,11,12,13,36
^ For example, Rostron et al^
36
^ reported a 10.89 times higher oral cancer mortality risk among males and a 5.08 times higher mortality risk among females who smoke cigarettes compared to those who never smoked cigarettes. In addition, cigarette smoking can cause dysregulation of immune responses, changes in the oral microbiome, and inhibit tissue repair,^
17,33,34
^ which increases the risk of periodontal diseases, a broad spectrum of conditions that encompasses gingivitis and periodontitis. Moreover, a recent review indicated that levels of proinflammatory cytokines, such as IL-1β, IL-6, interferon-γ, tumor necrosis factor α, and matrix metalloproteinase MMP-8 and MMP-9 in gingival crevicular fluid (GCF) were statistically significantly higher in AS compared to non-smokers.^
40
^ A systematic review of prospective studies estimated that smoking increases the risk of periodontitis by 85%.^
23
^ In addition, cigarette smoking is a significant predictor of tooth loss in patients with periodontitis.^
15
^ Chronic periodontitis can occur when untreated or unmanaged gingivitis progresses to the loss of the supporting tissues, which creates the deep periodontal pockets that are a hallmark of the disease and can eventually lead to tooth loss.^
25
^


Public health authorities, including the Food and Drug Administration (FDA), have acknowledged a continuum of risk across tobacco products, with combustible cigarettes at the highest end and non-combustible products, such as e-vapor, heat-not-burn, and oral tobacco products (e.g., nicotine pouches) on the lower end of the continuum.^
29,32
^ Oral tobacco-derived nicotine products such as nicotine pouches (for example on!® nicotine pouches, here referred to as the test product) are an emerging category of products that are tobacco-leaf—free and contain tobacco-derived nicotine, flavours, and excipients. Because these products are smoke-free and do not contain tobacco leaves, most of the harmful and potentially harmful chemicals are not detectable or are substantially reduced (by >99%).^
43
^ Moreover, a randomised controlled clinical study has demonstrated that AS switching from cigarettes to nicotine pouches (NP; specifically the test product), could substantially reduce their exposure to such chemicals,^
8
^ except nicotine. Nicotine pouches, including the test product, are not risk-free because they contain nicotine, which is addictive. Nonetheless, due to the substantial reduction in exposure to the toxins, adults who smoke and are unable or unwilling to quit all tobacco or nicotine-containing products may experience a reduction in harm if they completely switch to these products. To date, there is little research on the health effects of using nicotine pouches, including the test product, on oral health outcomes, which could be a cause of public health concern,^
37
^ especially since these products are typically placed under the upper lip. Therefore, the purpose of this study was to examine the potential impact on oral health among AS switching to the test product. We conducted this randomised controlled clinical study to characterise specific oral-health endpoints, primarily related to gingival inflammation, among AS who switched from conventional lit-end cigarettes to using the test product, compared to AS who simply continued cigarette smoking over 24 weeks.

## Materials and Methods

### Ethical Approval

This single-center study was designed and conducted following Good Clinical Practice (GCP) based on the current International Council for Harmonisation of Technical Requirements for Pharmaceuticals for Human Use (ICH) guidelines and the corresponding sections of Title 21 of the Code of Federal Regulations (CFR) Part 50 (Protection of Human Subjects), Title 21 CFR Part 56 (Institutional Review Boards [IRB]) and the Declaration of Helsinki. The relevant study documents (protocol, informed consent form [ICF], etc) were submitted to and were reviewed and approved by an independent IRB, Advarra Institutional Review Board (Columbia, MD, USA), prior to study initiation (IRB Approval # Pro00056024 dated July 26, 2021). Before conducting any study-specific procedures, all participants provided informed consent using the ICF per Title 21 CFR Part 50 governing the Protection of Human Subjects, local regulations, ICH guidelines, and the IRB or clinical trial center.

### Study Participants

This study enrolled adults (23–65 years of age) who had been smoking at least 10 combustible cigarettes per day regularly for at least 2 years before screening and were not planning to quit smoking in the next 30 days. Smoking status was biochemically confirmed through urinary cotinine levels (≥ 200 ng/ml at screening visit). Inclusion criteria required that: 1) all participants had a minimum of 18 ‘scorable’ teeth with scorable buccal and lingual surfaces; 2) generalised gingivitis characterised by the Modified Gingival Index (MGI) ≥ 1.75; 3) a Gingival Bleeding Index (BI) of ≥ 10 bleeding sites at both screening and baseline visits; and 4) agreed to stop using antiplaque/antigingivitis oral care products (mouthrinse and toothpastes) following the baseline visit through end of study (EOS). Key exclusion criteria were primarily focused on any clinically relevant medical condition that could jeopardise the safety of the participant or impact the validity of the study results, and included: 1) a planned postponement of a tobacco use cessation attempt in order to participate in the study or were planning on quitting smoking in the next 3 months from the baseline or screening; 2) used any tobacco or nicotine-containing products other than combustible cigarettes within 30 days prior to the screening visit; 3) presented with ≥ 30% of teeth with stage II – IV periodontitis according to the American Academy of Periodontology revised classification system for periodontal and peri-implant diseases and conditions^
41
^ at screening and/or baseline; 4) had more than three teeth with periodontal pocket depths measuring more than 5 mm; 5) had received periodontal treatment within 6 months or periodontal surgery within 3 years prior to baseline; 6) presented with extensive crown or bridge work, dental implants, and/or rampant decay; and 7) had orthodontic bands, wires or brackets with the exception of fixed lingual wires. All participants indicated they were willing to replace their cigarettes for 24 weeks with the mint-flavoured TP containing either 2, 4, or 8 mg nicotine.

### Clinical Safety

Study-participant health evaluations included physical exams, measurements of vital signs, including blood pressure and history of chronic disease. The physical exam, a 12-lead ECG, clinical laboratory assessments (clinical chemistry, hematology, urinalysis, and serology), vital signs measurements, a urine drug screen, an alcohol breath test, a serum pregnancy test (females only), and follicle-stimulating hormone (postmenopausal females only) tests. An oral soft- and hard-tissue (OSHT) exam was performed at baseline, week 12 and 24 visits. Adverse experiences (AEs), including findings from the OSHT exam, were monitored from the time of the first test product used until EOS or early termination, and were coded using the Medical Dictionary for Regulatory Activities (MedDRA v23.0).

### Study Products

The study products used here included participants’ usual brand (usually smoked by the participants and provided by the participants) cigarettes in the control group. The test product ([TPs] on!® nicotine pouches) is an oral pouched-tobacco product that contains pharmaceutical-grade tobacco-derived nicotine, microcrystalline cellulose, flavours, sodium carbonates and binders; they do not contain cut, ground, powdered, or leaf tobacco. The test product included three mint-flavoured (due to popularity in the marketplace) nicotine pouches at three nicotine levels: 2 mg, 4 mg, and 8 mg. Participants were directed to place the TP between the upper lip and gum, on whichever side of the mouth they chose.

### Study Design

This study used a randomised, controlled, open-label, 2-arm, parallel-group approach to evaluate changes in oral-health endpoints among adult smokers who switched from cigarette smoking to using TP compared to adults who just continued cigarette smoking (CS) over 24 weeks. Participants were screened within one month of the baseline visit. Upon completion of the baseline measurements, participants were stratified by age, gender, and BI and were randomly assigned to the test or control group (3:2 ratio).

Group 1: Test product group. Participants were instructed to discontinue cigarette smoking and instead use mint TP (any of the three strengths [2 mg, 4 mg and/or 8 mg] at participant’s choice) ad libitum, until the end of study (EOS) orGroup 2: CS group. Participants were to continue smoking their usual brand cigarettes until the EOS. 

Following the baseline visit, participants returned to the study site every two weeks to receive product supplies, and dental measurements were repeated at week 12 and week 24 visits. The participants in both groups were allowed the choice of quitting all tobacco products. The 6-month clinical study design was considered a reasonable study duration due to its frequency of use in dental research and because it is the current standard-of-care visit frequency recommended for individuals with gingivitis and Stage I periodontitis.^
41,42
^ The study duration is also consistent with the “FDA Draft Guidance for Industry, Gingivitis: Development and Evaluation of Drugs for Treatment or Prevention” (https://www.fda.gov/media/71536/download).

### Product Use Behaviour

All participants maintained a daily diary and self-recorded their use of test products per day (TPPD, test group only) or cigarette per day (CPD, both the test and control groups). For the test product group, the number of pouches used was documented in a dispensing log based on the difference between the quantity dispensed and the quantity of unused test products returned at each visit. Product-use behaviour was assessed by mean CPD and mean TPPD, which were recorded in the participant’s diary and through the dispensing log. Participants in each study group were requested to report any other types of nicotine/tobacco product use during the study. Urinary total 4-[methylnitrosamino]-1-[3-pyridyl]-1-butanol (total NNAL) were measured at baseline, weeks 12 and 24 as a study-product compliance check.

### Study Endpoints

The primary endpoints were the mean changes from baseline in full-mouth MGI and BI at 24 weeks. The mean change in full-mouth MGI and BI at 12 weeks from baseline was assessed as a secondary endpoint. Other secondary endpoints included the mean changes from baseline to 12 and 24 weeks for a composite value of the Lobene Stain Index (LSI), Turesky Plaque Index (TPI), Periodontal Probing Depth (PPD), Bleeding on Probing (BOP) and Gingival Crevicular Fluid (GCF) volume. These endpoints are dental indices for measuring the status of gingivitis and bleeding and are standard dental clinical diagnostic tools routinely used in dental practice to assess the status of gingivitis and periodontitis.^
21,22
^ Definitions and scores for MGI, BI, LSI, and TPI to assess gingival inflammation, gingival bleeding tendency, stain index, and plaque are provided in the Appendix. Full-mouth PPD measurements were performed on all scorable teeth and were measured to the nearest millimeter, recorded as the distance from the gingival margin to the base of the pocket. Full-mouth PPD was calculated for each participant by adding the individual PPD measurements and dividing this sum by the number of sites measured. After measuring the PPD, the corresponding sites were inspected for the presence or absence of bleeding. If bleeding occurred, a positive finding was recorded, and the result was expressed as the number of positive sites and as a percentage of the number of sites examined. Assessments of these dental endpoints were conducted by three clinical examiners who were blinded to the study assignment and had at least 5 years of experience in the use of these indices. The same examiner scored the same index throughout the study. Examiner repeatability was achieved by showing > 85% agreement (Pearson’s correlation coefficient r = 0.999 for MGI and r = 0.989 for PI) between repeat assessments, prior to initiation of the study. GCF samples were collected at the mesial buccal sites of two different teeth. A gentle air stream was directed towards the tooth surface for 5 s to dry the area. GCF was collected by inserting a Perio-paper strip (IDE Interstate; Amityville, NY, USA) into the gingival crevice and left in place for 30 s. Immediately after collection, the GCF volume was measured by Periotron 8000 (Oraflow; Smithtown, NY, USA). We also collected subgingival plaque samples for microbiome analyses; the results from the microbiome findings will be reported separately.

### Sample Size Estimation

The purpose of this study was to characterise the changes in oral health outcomes. This study intended to investigate these outcomes among adult smokers who switched to nicotine pouches. Due to the paucity of published data on the effect of nicotine pouches (including the test product) on chronic periodontitis or gingivitis, we did not define a hypothesis. The findings from this study could be used to definitively demonstrate with the hypothesis that switching from cigarettes to nicotine pouches would not increase gingival inflammation. No formal sample size estimation was performed in this study. There is limited published data on the effect of nicotine pouches, including the test product, on chronic periodontitis or gingivitis, and the changes among AS switching to noncombustible tobacco products for the endpoints measured. Based on a review of the literature, the typical sample size of studies evaluating oral health outcome measures among smokers have ranged from 22 to 30.^
6,19
^ Therefore, the sample size (n = 150) included in this study was considered sufficient to characterise the impact of using the test product over 24 weeks for the proposed endpoints compared to continued smoking.

### Statistical Analysis

The endpoints in this study were assessed comparing the mean between-group changes from baseline to week 12 and baseline to week 24. Product-use behaviours were summarised using descriptive statistics. For dental endpoints, a linear mixed model analysis for repeated measurements (MMRM) included the change from baseline as the dependent variable, study group, gender, BI category, and age category as fixed effects, and the baseline score as a covariate. Using the SAS software version 9.4 (SAS, Cary, NC, USA) procedure PROC MIXED, the difference in least-squares means was used for comparisons, and p-values and two-sided confidence intervals were calculated for the mean change from baseline for each study group, as well as the comparison of these changes between study groups. The latter was used for statistical significance determination for the difference between test product and control at each post-baseline time point. The intention to treat (ITT) population included every participant who was enrolled and randomised according to the randomisation schedule, while the modified intention to treat (MITT) population included those participants in the ITT population for whom there was a baseline and at least one post-baseline dental measurement. Unless otherwise specified, all analyses of study endpoints presented were performed on the MITT populations. The MITT population was further divided into two subsets as “on!® switchers subset (OSS)” and the “dual-use subset (DUS)”. The OSS included those participants who exhibited adherence to the product use requirements for the test group (i.e., switching from cigarette smoking to the use of the test product as defined by smoking 10% or less of the baseline CPD with total NNAL level being 25% or less of the baseline level). The DUS included those in the test product group who reported product use but failed any one of the criteria for OSS.

## Results

The underlying data related to this study will be shared on reasonable request to the corresponding author.

### Participant Demographics 

**Fig 1 fig1:**
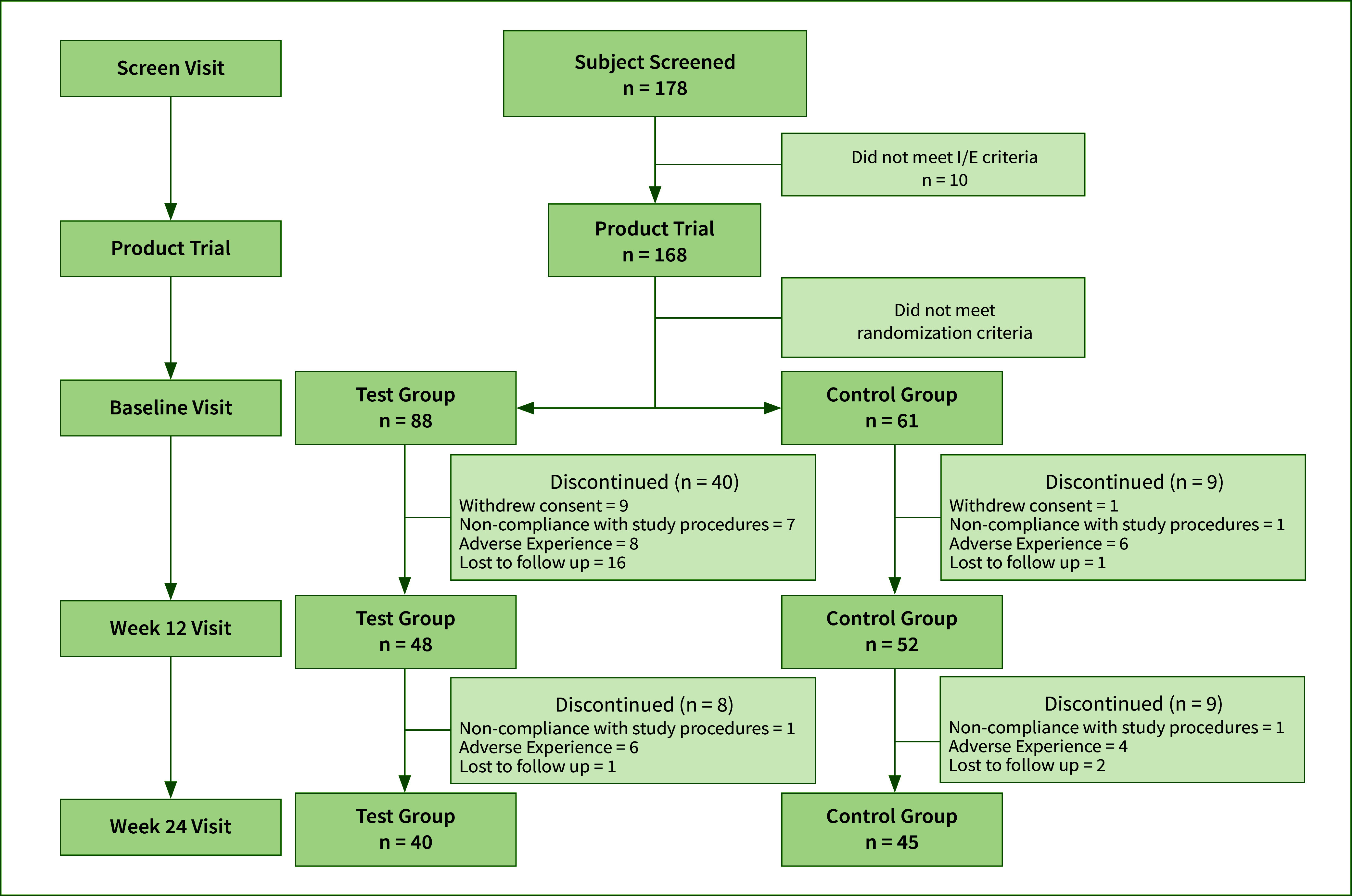
Flowchart of participant selection and group assignment.

Overall, participants’ demographic distribution was generally consistent between the control and test groups (Table 1). Among the AS screened for participation in the study (n = 178), 149 were randomised, 100 were enrolled and completed week 12 (MITT population), and 85 completed week 24 events (Fig 1). Subject ages ranged from 22 to 63 years old, with a mean age of 41.2 years. The distribution in the CS and TP groups were similar with a mean age of 39.8 (SD 10.71) and 40.5 (SD 11.38), respectively. The majority of the enrolled participants were female (n = 56, 56.0%) and White (n = 78, 78.0%) and were comparable between the two study groups (CS: 52.1% female, 79.2% White; TP: 59.6% female, 76.9% White). Black or African Americans made up 20% of the overall population and the proportions were similar in the CS (21.2%) and TP group (18.8%). The majority of participants indicated that they were not Hispanic nor Latino (overall: 93%, CS: 92.3%, TP: 93.8%). The smoking history was 21.6 years and 17.8 years for the CS and TP groups, respectively, with a similar number of CPD between the CS and TP groups (median of 15 cigarettes per day).

**Table 1 table1:** Participant population and demographics

	Test group (n = 48)	Control group (n = 52)	Overall (n = 100)
**Age in years**
Mean (SD)	39.8 (10.71)	42.4 (11.38)	41.2 (11.09)
Median	39.0	40.5	39.5
Min, Max	22, 60	23, 63	22, 63
**Gender, n (%)**
Male	23 (47.9%)	21 (40.4%)	44 (44.0%)
Female	25 (52.1%)	31 (59.6%)	56 (56.0%)
Race, n (%)
White	38 (79.2%)	40 (76.9%)	78 (78.0%)
Black or African American	9 (18.8%)	11 (21.2%)	20 (20.0%)
Black or African American, White	1 (2.1%)	1 (1.9%)	2 (2.0%)
Asian	0	0	0
American Indian or Alaska Native	0	0	0
Ethnicity, n (%)
Hispanic or Latino	3 (6.3%)	4 (7.7%)	7 (7.0%)
Not Hispanic or Latino	45 (93.8%)	48 (92.3%)	93 (93.0%)
Years smoked cigarettes
Mean (SD)	17.8 (11.39)	21.6 (12.08)	19.8 (11.85)
Median	15.0	20.0	20.0
Min, Max	3, 40	2, 50	2, 50
Cigarettes per day
Mean (SD)	15.4 (4.86)	17.5 (9.58)	16.5 (7.72)
Median	15.0	15.0	15.0
Min, Max	10, 30	10, 60	10, 60


### Product Use and Compliance

**Fig 2 fig2:**
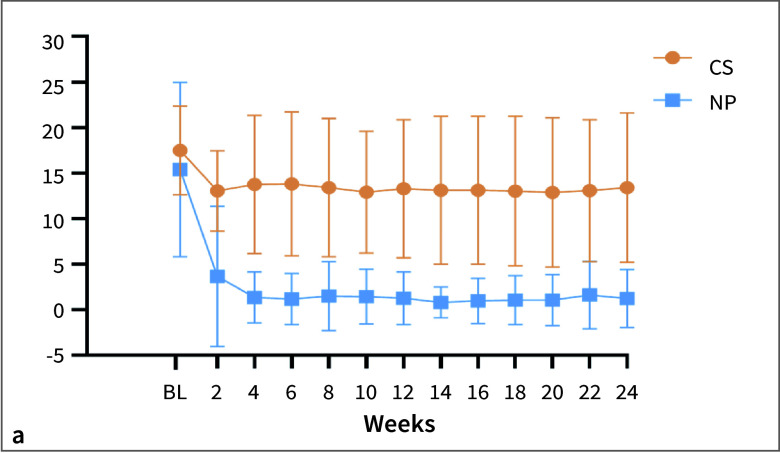

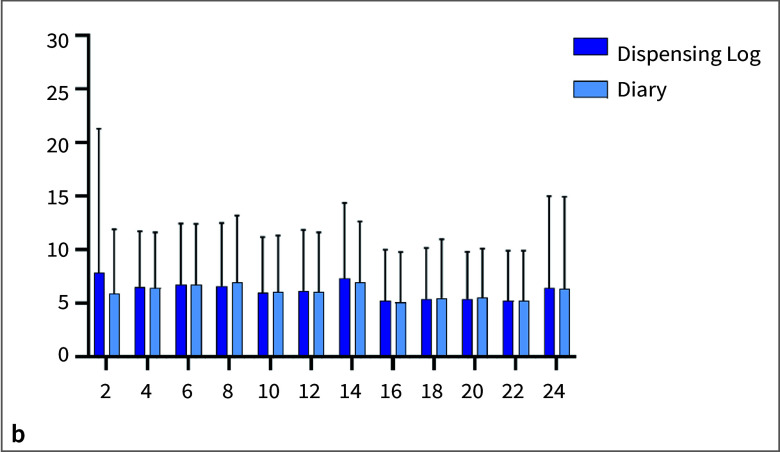
(a) Summary of daily cigarette and on!® nicotine pouch use (mean ± SD) reported at bi-weekly visits, based on the daily diary entry used to record the number of cigarette and nicotine pouch use. (b) Verification of self-reported values against the products dispensed at the clinic site. The dispensing log was based on the number of nicotine pouches calculated by subtracting the number returned from the number dispensed as recorded in the dispensing log at each visit. NP = on!® nicotine pouch; BL = baseline.

Before randomisation, participants smoked 15.4 to 17.5 cigarettes on average, for the TP and CS groups, respectively. Daily cigarette use dropped by 1 to 2 cigarettes for participants in the CS group while participants in the TP group reduced their cigarette consumption on average to ~1 per day through both weeks 12 and 24 (Fig 2a). Participants in the TP group used an average of 5 to 7 pouches per day (Fig 2b). TP use as reported by the participants’ diaries aligned with the TP dispensed logs. Four participants in the TP group were withdrawn due to non-compliance with the use of study products. The participation of 5 individuals (4 in the TP and 1 in the CS group) was discontinued because of non-compliance with study procedures.

### Primary Endpoint

#### Modified Gingival Index and Bleeding Index

**Fig 3 Fig3:**
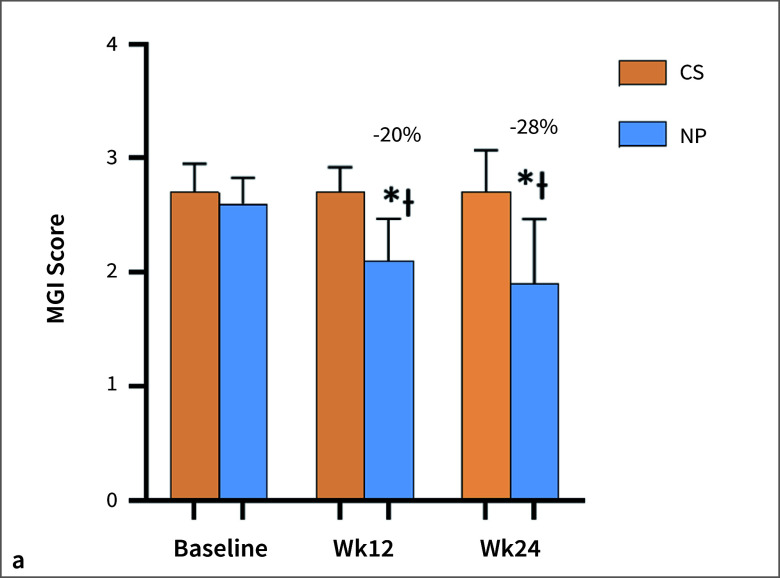

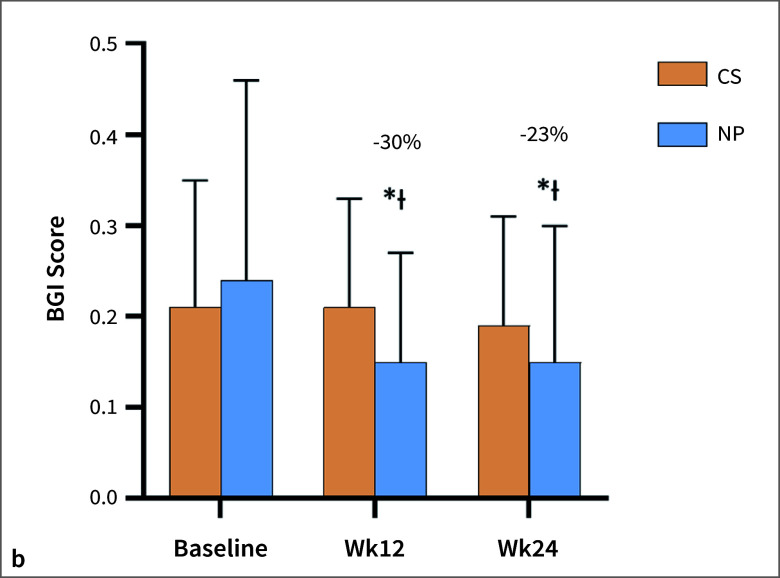

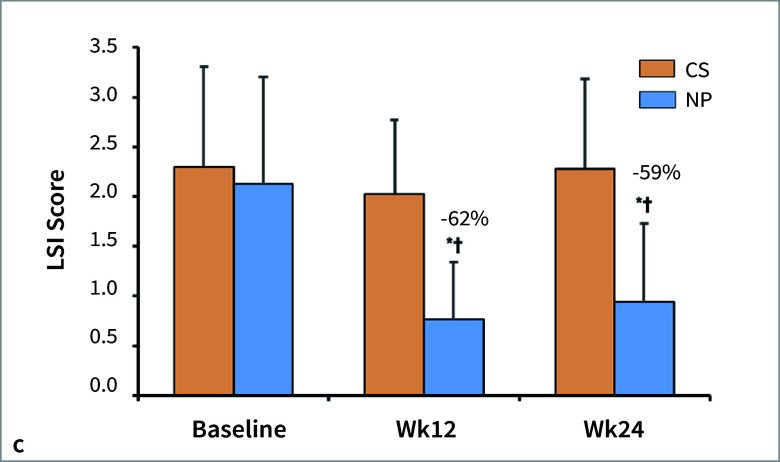
Summary of primary oral health endpoints (mean ± SD). (a) Modified Gingival Index (MGI); (b) Bleeding Index (BI); and (c) Lobene Stain Index (LSI) at baseline (BL) and weeks 12 and 24. The percent reduction = percentage difference between mean values of the NP (on!® nicotine pouch) group vs the continue smoking (CS) Group mean at the corresponding visit (reported for post-baseline). *p < 0.0001 compared to baseline; ^Ɨ^p < 0.0001 changes from baseline compared to the CS group.

Compared to the CS group, there was a statistically significant reduction in full-mouth MGI scores at weeks 12 and 24 for the TP group (overall average reduction of 20% and 28%, respectively, p  <  0.001 (Fig 3a). Similar observations were seen in the TP group for the BI scores compared to the CS group at both week 12 and week 24 (overall average reduction of 30% and 23%, respectively, p  <  0.05, Fig 3b). A statistically significant reduction in gingival inflammation scores was also observed in the TP group at week 24, when compared with the CS group (28.4% [p  <  0.0001] and 22.8% [p = 0.0078] for both MGI and BI, respectively). The changes in these endpoints for the OSS and DUS were generally comparable (Supplemental Tables S1 and S2). On the other hand, no statistically significant differences were observed for any of these endpoints in the CS group at week 24 compared to baseline.

### Secondary Endpoints

#### Lobene stain index

Statistically significant reductions in composite stain scores were seen for participants of the TP group at both week 12 and 24 compared to the CS group (p  <  0.001). Composite stain scores for the TP group at week 12 and week 24 were 62% and 59% lower, respectively, than the CS group (Fig 3c). Similar observations were noted for the OSS and DUS groups (Supplemental Table S3).

#### Plaque index

There were no statistically significant changes from baseline for the TP or CS groups, or any statistically significant differences between the TP and the CS groups at any time point.

#### Periodontal probing depth (PPD) and bleeding on probing (BOP)

No statistically significant differences in PPD were observed compared to baseline at week 12 and week 24 for either the TP or CS groups, or between the two groups at the two timepoints. Furthermore, there were no statistically significant changes observed for the TP subsets (OSS and DUS) compared to the baseline or the CS group.

Similarly, the BOP values were not statistically significantly different between the two groups at week 12 and week 24. While there was no statistically significant difference between baseline and either week 12 or 24 for the TP group or between baseline and week 12 for the CS group, we did observe a statistically significant difference from baseline for the CS group at week 24. The least-squares mean (SE) difference of change from baseline in BOP for the CS group at week 24 was -0.029 (0.0101), which was statistically significantly different (p = 0.0052). Furthermore, there were no statistically significant changes observed in the BOP endpoint for the TP subsets (OSS and DUS) compared to the baseline or to the CS group at any post-baseline time points.

#### Gingival crevicular fluid volume

Statistically significant reductions (p  <  0.05) from the baseline in mean GCF volume were shown for the TP (-17.3 ul at week 12 and -20ul at week 24) and the CS (-14 µl at week 12 and -9.9 µl at week 24) groups. No statistically significant difference was observed between TP and CS groups at any time point (Supplemental Table S4).

### Clinical Safety

There were no deaths or serious adverse events during the study. There were 38 total AEs reported during this study among 33 participants, 23 for the TP and 15 for the CS group. Of the 38 AEs, 22 AEs were attributed to a positive SARS-CoV-2 test, i.e., 12 in the TP and 10 in the CS groups. The participation of these individuals in this study was discontinued. Of the 16 remaining AEs, nine were not classified by the clinic staff as product related. Seven AEs reported among 5/88 participants in the TP group were considered product related (likely, probably, or definitely) which were resolved prior to the end of the study. These AEs included one each of aphthous ulcer (canker sore, mild), dysphagia (mild), gingival discomfort (moderate), headache (moderate), cough and oral discomfort (mild). Two participants withdrew from the study due to the AEs: one due to cough and dysphagia (mild), and the second participant withdrew due to gingival discomfort (moderate) and headache (moderate).

## Discussion

This randomised controlled study demonstrates that AS using the TP over 24 weeks showed statistically significantly reduced gingival inflammation, as measured by MGI and BI, compared to those who continued to smoke cigarettes. Despite indicating no intention to quit smoking in the next 30 days, a substantial reduction in average cigarette consumption (> 90%) was observed in the TP group. We observed reductions of 28% in MGI and 23% in BI scores in the TP group compared to the CS group at week 24. No changes in gingival inflammation endpoints were observed in the CS group compared to baseline. Overall, the findings reported in this study suggest that AS who switch to the TP may reduce their risks of gingivitis and the deterioration of periodontal health relative to continued smoking. These findings are most likely due to the substantial reduction in cigarette consumption, thereby reducing exposure to many of the toxins present in cigarette smoke.

Cigarette smoking facilitates the development and progression of periodontal diseases, even when accounting for other contributory factors, such as oral hygiene, plaque, calculus, and socioeconomic status and demographic differences. The odds ratio of developing periodontal diseases has been estimated to be 2.5 to 6.0 times higher in smokers with an apparent dose-response relationship with smoking intensity.^
3
^ For example, the probability for further tooth attachment loss ranges from 2.05 for light smokers to 4.75 for heavy smokers.^
1
^ Gingivitis is a precursor to periodontitis,^
20,27
^ and the MGI^
24
^ is an established method of assessing gingival health. In this study, most of the AS had baseline MGI scores indicative of generally moderate inflammation (average median score ~3), which statistically significantly decreased to mild inflammation (average median score ~2) after 12 and 24 weeks in the TP group, while remaining stable in the CS group. Although the reductions in the TP group were statistically significant, the clinical relevance of these findings remains to be established. The median baseline BI values among AS were reduced from ~15% to 10% by week 12 and to 8% by week 24 in the TP group, while remaining relatively constant in the CS group. Gingival bleeding is an objective, easily assessed sign of inflammation that is associated with periodontal disease. The most accepted index for measuring the prevalence of gingivitis is the presence, extent, or severity of bleeding.^
21,22
^ Thus, the observations related to BI appear to corroborate the findings regarding MGI. Our observations regarding the reduction of MGI align with a recent report of significant reduction in gingival inflammation as evaluated using the gingival index after six months of smoking cessation.^
34
^ However, our observations of reduction in BI are contrary to the reports of increases in gingival bleeding after three months of smoking cessation,^
27
^ which the authors propose as being likely due to the removal of the vasoconstrictive effects of nicotine from smoking cessation. The vasoconstrictive effects of nicotine may not be inconsequential. Luzzi et al^
25
^ indicated that cigarette smoking can result in “small, chronic and repetitive vasoconstrictive attacks”, as well as revascularization impairment. Newbrun^
33
^ also attributed the lower gingival bleeding tendency in tobacco users to the repeated vasoconstrictive effects of nicotine. Additionally, during our exploratory assessment of the oral microbiome in the subgingival plaque, some evidence suggested that the species of bacteria in plaque changed among AS completely switching to the TP, compared to those who continued smoking.^
35
^ Therefore, it is possible that our observations of MGI and BI improvements in smokers switching to the nicotine pouches are due to changes in the immune responses and in the bacterial species in the subgingival plaque while maintaining nicotine exposure. Collectively, the two endpoints, MGI and BI, indicate that gingival inflammation was lower among AS using the TP.

Changes in the other endpoints, PI, BOP, and PPD, were not observed in the TP or CS group. It is noteworthy that the MGI reduction was not accompanied by a concurrent reduction in PI, which may appear to be counterintuitive, since the two end-points are closely associated.^
38
^ In the current study, at baseline, all subjects received full prophylaxis (all tartar, stain, and plaque removed) and standardised oral hygiene material (toothbrush and toothpaste) along with instructions to use them. This probably presented an opportunity for oral health improvement in both the CS and TP groups. The baseline prophylaxis of plaque removal, along with heightened awareness of maintaining oral hygiene due to regular visits to the dental clinic, could have resulted in comparable levels of PI in the CS and TP groups. This may account for the lack of measurable changes in the PI outcome between the two groups. Furthermore, PI is highly variable. The US Surgeon General’s report describes that although some studies found smokers to have more visible bacterial plaque than nonsmokers, many other studies reported no statistically significant differences in mean plaque levels or rates of plaque accumulation.^
30
^ The presence of specific bacterial species in the plaque may be more important than the quantity of visible plaque and debris on the teeth in the pathogenesis of severe periodontitis,^
14
^ and smokers may be more likely than nonsmokers to harbor specific periodontal pathogens.^
44
^ Therefore, it is likely that changes in the composition of specific bacterial species in the plaque and not the amount may account for the reduction in MGI despite no changes in the PI outcome in our study.

Although no statistically significant difference was observed between the TP and CS groups in mean GCF volume, mean GCF volume from both TP and CS groups at week 12 declined by 17 µl for TP (p < 0.05) and 14 µl for CS, and by 20 µL (TP) and 10 µL (CS) at week 24. The clinical relevance of this reduction of ~18% is unclear. These observations align with the lack of statistically significant differences between the mean GCF volume between smokers and nonsmokers, which has been reported elsewhere.^
7
^


We compare our findings with other emerging non-combustible products, specifically e-cigarettes. In a recent systematic review on impact of e-cigarettes and conventional cigarettes on periodontal health, Thiem et al^
40
^ conducted a meta-analysis based on seven studies. The authors report a statistically significantly lower likelihood of BOP among e-cigarette users compared to cigarette smokers. However, we did not observe differences in BOP between the CS and TP groups. These differences could be due to different study designs: our study was a randomised controlled clinical study, whereas that of Thiem et al^
40
^ was based three case-control studies, three cross-sectional study, and one cohort study. Due to differences in study designs, direct comparisons of literature reports to the findings of the present study regarding the BOP results may not be possible. Another reason for the differences could be attributed to the inclusion/exclusion criteria as stated in the “Materials and Methods” section. For example, we excluded participants with advanced periodontitis (stage II-IV), so that the impact of switching to the TP may not be as profound for some of the endpoints. Additionally, the authors note that due to differences in study populations and clinical assessments as well as self-reports from patients or non-standardised measures of bleeding indices and PI, mixed results have been reported. Holliday et al^
16
^ evaluated the literature on the impact of e-cigarettes on oral health and concluded that the totality of evidence suggests that the risk of periodontal disease associated with e-cigarette use is less than that associated with tobacco smoking, but more than that seen in nonsmokers. Those authors further conclude that, although it is limited and challenging to interpret, the clinical evidence suggests that e-cigarettes are less harmful to oral health than tobacco cigarettes, and might be an effective cessation aid in dental settings.

To the best of our knowledge, there are no studies that have evaluated the impact of smoking cessation on the endpoints used in this study. Mittal et al^
28
^ reported statistically significant increases in gingival bleeding and an increase in pocket depth after stopping smoking for 3 months,^
28
^ likely due to the removal of nicotine exposure. As a predictor of periodontal disease progression, bleeding on probing has low sensitivity owing to a high frequency of false-positive responses; however, it has high specificity in that failure to bleed indicates healthy tissue.^
31
^ There is evidence that smokers have less, or delayed, gingival bleeding when compared with non-smokers, likely due to the vasoconstrictive effects of nicotine.^
31
^ The lack of any observed increases in these endpoints in the present study may be accounted for by the potential localised vasoconstrictive effects from the nicotine released in the buccal region by the TP. Nonetheless, in a systematic review of cross-sectional and longitudinal studies, smoking cessation has a positive effect on periodontal disease, and the risk thereof reverses to never-smoker levels within a few years.^
10
^


Lastly, a substantial reduction in staining (~70%) was observed through the LSI endpoint among AS using the TP. These observations corroborate other published reports with other non-combustible tobacco products, such as heated-tobacco products.^
5
^ Visible discolouration from cigarette smoking is well-documented,^
18
^ which may result in social challenges among AS due to its deleterious effect on an individual’s appearance. Thus, AS may be motivated to switch to the TP to minimise this social unease and, in the process, reduce the harmful effects of continued smoking.

The findings from this study should be considered in the context of some limitations. This randomised study included a relatively large sample size of adults who continued to smoke (n = 52) and those who used the TP (n = 40-48) at the end of study. However, the number of participants who had biochemical verification of complete smoking cessation was relatively small. However, the participants who used the TP reduced their cigarette consumption by > 90%, so that the findings among the overall TP group may generally reflect complete switching to the TP. Moreover, due to the comparability of statistically significant reductions in the primary endpoints for both the overall TP and complete switcher (OSS) group, the findings may be considered applicable to the impact of complete switching to TP. The inclusion/exclusion criteria might be viewed as a limitation as well. For example, we excluded participants exhibiting ≥ 30% of teeth with stage II-IV periodontitis. We intended to include participants in the study who had a sufficient indication of gingivitis but had not yet progressed to overt periodontal disease, such that the potential impact of switching on gingival health could be discerned within the duration and conditions of the study.

The 24-week duration of the study might be viewed as a limitation regarding the long-term health effects of the TP. However, the 24-week duration can be considered adequate to determine potential long-term impact, given the lack of any remarkable differences between weeks 12 and 24. Furthermore, conducting the study beyond 24 weeks could likely yield a greater reduction but not change the conclusions regarding statisticaly significant reduction in the primary endpoints.

## CONCLUSIONS

Undoubtedly, quitting smoking is the best option for those AS who are concerned about the health effects of smoking. However, for those individuals unable or unwilling to quit using tobacco or nicotine-containing products, switching to a nicotine pouch product like the TP will substantially reduce exposure to many of the toxins,^
8
^ which should reduce the risk of smoking-related diseases, including periodontal disease.

Due to the emerging growth of nicotine pouches,^
26
^ there is growing recognition among public health researchers for the need for studies to elucidate the impact of such products on periodontal health^
26
^ as well as any other potential side-effects.^
9
^ We present compelling evidence regarding reduction in gingival inflammation (an early indicator of periodontal disease as defined by standardised dental indices) among AS who had either switched to the TP or substantially reduced cigarette consumption. For the primary endpoint analysis, significant reductions in gingival inflammation and bleeding (MGI and BI) were observed in AS who switched to ad libitum use of the TP for 24 weeks compared to AS who continued smoking for 24 weeks. The clinical relevance of these findings remains to be established. However, within the context of the limitations and under the study conditions, our findings suggest that the use of the TP does not result in further deterioration of the periodontal health of AS using the TP. The results from this study demonstrate that the TP (on!® nicotine pouches), while not risk-free, have the potential to reduce harm among AS who are unable or unwilling to quit smoking cigarettes.

## Acknowlegdements

Scientific writing support was provided by the Editorial Services Team at Altria. The authors are grateful to all the participants who devoted their time and effort in support of this study. This study was fully funded by Altria Client Services LLC., Richmond, VA USA.

## Conflict of Interest

Jianmin Liu, Jingzhu Wang, Jeffery S. Edmiston, Mohamadi A. Sarkar, Maria Gogova are employees of Altria Client Services LLC., Richmond, VA, USA; Kimberly R. Milleman, Jeffery L. Milleman, Abigale L. Yoder, are employees of Salus Research, Inc., Fort Wayne, IN, USA.

## References

## APPENDIX

### Supplemental Information 1, Definition of study endpoints measured at baseline, week 12 and week 24 visits

### Modified Gingival Inflammation (MGI)

At baseline, week 12 and week 24 visits, gingival inflammation was assessed according to MGI. The method is a non-invasive assessment of visual changes in severity and extent of gingival inflammation. Each of the six gingival areas (distofacial, facial, mesiofacial, distolingual, lingual and mesiolingual) of all scorable teeth were evaluated using a scale of 0 – 4 as noted below:

0 = Normal (absence of inflammation).1 = Mild inflammation (slight change in colour, little change in texture) of any portion of the entire gingival unit.2 = Mild inflammation of the entire gingival unit.3 = Moderate inflammation (moderate glazing, redness, oedema, and/or hypertrophy) of the gingival unit.4 = Severe inflammation (marked redness and oedema/hypertrophy, spontaneous bleeding, or ulceration) of the gingival unit.

Full-mouth MGI scores were calculated by summing all scores and dividing by the number of scorable sites examined.

### Bleeding Index (BI)

Gingival bleeding tendency was assessed according to the BI at baseline, week 12 and week 24 visits. A periodontal probe was inserted into the gingival crevice to a depth of approximately 1 mm and moved gently around the tooth, stroking the inner surface of the sulcular epithelium. Each of six gingival areas of each tooth (distobuccal, midbuccal, mesiobuccal, distolingual, mesiolingual, and midlingual) was probed in a likewise manner, waiting approximately 30 seconds before recording the number of gingival units which bleed, according to the following scale:

0 = absence of bleeding after 30 seconds.1 = bleeding observed after 30 seconds; and2 = immediate bleeding observed.

The full-mouth BI score for each subject was calculated as the mean BI over all tooth sites.

### Lobene Stain Index (LSI)

Extrinsic tooth stain was measured at baseline, week 12 and week 24, according to the LSI on the facial and lingual surfaces of up to 12 anterior teeth, #6 – 11 and #22 – 27. Non-scorable and missing sites were not included in the statistical analyses. Each facial and lingual surface of each anterior tooth was divided into the gingival region and the body region.

The intensity and area (extent) of stain in each of the two tooth regions is assessed using 0 – 3 scales:

The Lobene Stain Index is calculated for each subject by averaging the intensity score (sum of all intensity scores/all sites graded), the extent score (sum of all extent scores/all sites graded), and the product (composite) of the two scores (sum of all intensity x extent scores/all sites graded). In addition to the stain area and stain intensity score, a composite score is obtained for each surface by multiplying the area score by the intensity score.

### Clinical Dental Endpoints

For each of the dental endpoint scores, the subject-wise change from the value obtained at the baseline visit was calculated for both the week 12 visit and the week 24 visit.

Primary study endpoints based on the clinical dental assessments were:

Change from baseline MGI at the week 24 visit.Change from baseline BI at the week 24 visit.

Secondary study endpoints based on the clinical dental assessments were:

Change from baseline MGI at the week 12 visit.Change from baseline BI at the week 12 visit.Change from baseline BOP at the week 12 and week 24 visits.Change from baseline LSI at the week 12 and week 24 visits.

For LSI, which is only evaluated on the facial and lingual surfaces of anterior teeth, the associated endpoints were limited to assessments made on facial sites, and those made on lingual sites. In addition to the stain area and stain intensity score, a composite score was obtained for each surface by multiplying the area score by the intensity score.

For all the study endpoints, data summaries were provided by study group for the scores obtained at each visit (including screening for MGI and BI), and for the changes from baseline at the week 12 and week 24 visits.

For the dental endpoints, the mean changes from baseline at week 12 and at week 24 were compared between study groups employing a linear analysis for MMRM that included the change from baseline as the dependent variable; study group, gender, BI category and age-category as fixed effects; and the baseline score as a covariate. The SAS procedure PROC MIXED was employed, and the difference in least-squares means was used for comparisons, accompanied by the p-values and two-sided confidence intervals both for the mean change from baseline for each study group, and for the comparison of these changes between study groups (presented as percent changes from the control group). The data for the clinical dental assessments were presented in listings.

## Supplemental information 2

**Table d67e1057:** 

Stain area	Stain intensity
0 = No stain detected;	0 = No stain;
1 = Stain up to one-third of the region;	1 = Light stain – yellow/tan;
2 = Stain up to two-thirds of the region;	2 = Moderate stain – medium brown;
3 = Stain over more than two-thirds of the region.	3 = Heavy stain – dark brown/black

**Supplemental Table S2 SupplementaltableS2:** Summary of bleeding index scores

	Summary of scores at visit	Summary of changes from baseline
n	Mean (SD)	% Reduction vs control^‡^	n	Mean (SD)	p-value vs baseline*	p-value vs control^†^
MITT population							
Test group overall							
Baseline	48	0.208 (0.1671)	–				
Week 12	48	0.148 (0.1190)	29.8%	48	-0.060 (0.1227)	< 0.0001	< 0.0001
Week 24	40	0.151 (0.1515)	22.8%	40	-0.057 (0.1049)	0.0009	0.0078
OSS subset							
Week 12	12	0.150 (0.1361)	28.5%	12	-0.059 (0.1090)	0.0213	0.0258
Week 24	10	0.159 (0.1302)	18.6%	10	-0.046 (0.1321)	0.0967	0.1936
DUS subset							
Week 12	31	0.154 (0.1215)	26.9%	31	-0.071 (0.1351)	0.0004	0.0016
Week 24	26	0.148 (0.1681)	24.6%	26	-0.067 (0.1007)	0.0011	0.0099
Control group							
Baseline	52	0.180 (0.0998)	n/a				
Week 12	52	0.210 (0.1177)	n/a	52	0.030 (0.1028)	0.2418	n/a
Week 24	45	0.196 (0.1215)	n/a	45	0.019 (0.1031)	0.8748	n/a
‡% Reduction = percentage difference of test group means vs the control group mean at the corresponding visit (reported for post-baseline visits only). A positive value of the % difference reflects a lower score for the test group being summarised. *Within-group p-value comparing the mean score at the follow-up visit vs the mean score at baseline. †Between-group p-value comparing the mean change from baseline for the indicated test group vs the corresponding change for the control group. MITT = Modified Intent to Treat; OSS = on!® switchers subset; DUS = dual-use subset.

**Supplemental Table S1 SupplementaltableS1:** Summary of Modified Gingival Index scores

	Summary of scores at visit	Summary of changes from baseline
n	Mean (SD)	% Reduction vs control ‡	n	Mean (SD)	p-value vs baseline*	p-value vs control†
MITT population							
Test group overall							
Baseline	48	2.637 (0.2398)	--				
Week 12	48	2.187 (0.3714)	20.3%	48	-0.451 (0.2766)	< 0.0001	< 0.0001
Week 24	40	1.920 (0.5652)	28.4%	40	-0.709 (0.4196)	< 0.0001	< 0.0001
OSS subset							
Week 12	12	1.960 (0.1854)	28.6%	12	-0.660 (0.2298)	< 0.0001	< 0.0001
Week 24	10	1.799 (0.4083)	33.0%	10	-0.741 (0.3544)	< 0.0001	< 0.0001
DUS subset							
Week 12	31	2.258 (0.4016)	17.7%	31	-0.401 (0.2663)	< 0.0001	< 0.0001
Week 24	26	1.916 (0.5834)	28.6%	26	-0.739 (0.4227)	< 0.0001	< 0.0001
Control group							
Baseline	52	2.702 (0.2514)	n/a				
Week 12	52	2.744 (0.2182)	n/a	52	0.042 (0.1769)	0.0406	n/a
Week 24	45	2.683 (0.3717)	n/a	45	-0.011 (0.3754)	0.8481	n/a
‡% reduction = percentage difference of test group means vs the control group mean at the corresponding visit (reported for post-baseline visits only). A positive value of the % difference reflects a lower score for the test group being summarised. *Within-group p-value comparing the mean score at the follow-up visit vs the mean score at baseline. †Between-group p-value comparing the mean change from baseline for the indicated test group vs the corresponding change for the control group. OSS = on!® switchers subset; DUS = dual-use subset.

**Supplemental Table S3 SupplementaltableS3:** Summary of LSI composite scores

	Summary of scores at visit	Summary of changes from baseline
n	Mean (SD)	%Reduction vs control^‡^	n	Mean (SD)	p-value vs baseline*	p-value vs control^†^
MITT Population							
Test group overall							
Baseline	48	2.125 (1.0799)	--				
Week 12	48	0.764 (0.5775)	62.3%	48	-1.361 (0.8217)	< 0.0001	< 0.0001
Week 24	40	0.943 (0.7799)	58.7%	40	-1.240 (0.8949)	< 0.0001	< 0.0001
OSS subset							
Week 12	12	0.653 (0.5943)	67.8%	12	-1.358 (1.0250)	< 0.0001	< 0.0001
Week 24	10	0.624 (0.3469)	72.6%	10	-1.449 (0.9881)	< 0.0001	< 0.0001
DUS subset							
Week 12	31	0.783 (0.5612)	61.3%	31	-1.416 (0.7529)	< 0.0001	< 0.0001
Week 24	26	1.013 (0.8349)	55.6%	26	-1.295 (0.8321)	< 0.0001	< 0.0001
Control group							
Baseline	52	2.297 (1.0109)	n/a				
Week 12	52	2.025 (0.7458)	n/a	52	-0.272 (0.6152)	0.0011	n/a
Week 24	45	2.280 (0.9002)	n/a	45	0.024 (0.8275)	0.6863	n/a
‡ % Reduction = percentage difference of test group mean vs the control group mean at the corresponding visit mean for the control group (reported for post-baseline visits only). A positive value of the % difference reflects a lower score for the test group being summarised. * Within-group p-value comparing the mean score at the follow-up visit vs the mean score at baseline. † Between-group p-value comparing the mean change from baseline for the indicated test group vs the corresponding change for the control group. MITT = Modified Intent to Treat; OSS = on!® switchers subset; DUS = dual-use subset.

**Supplemental Table SupplementalTable:** S4 Summary of gingival crevicular fluid volume (µL)

	Summary of scores at visit	Summary of changes from baseline
n	Mean (SD)	n	Mean (SD)	p-value vs baseline*	p-value vs control^†^
MITT Population						
Test group overall						
Baseline	48	117.302 (33.5007)				
Week 12	48	100.031 (38.5645)	48	-17.271 (28.9941)	0.0004	0.4850
Week 24	40	97.875 (40.2980)	40	-20.775 (32.7155)	< 0.0001	0.1201
OSS subset						
Week 12	12	87.250 (32.7633)	12	-19.083 (31.9081)	< 0.0001	0.4831
Week 24	10	90.000 (33.6312)	10	-19.550 (28.5477)	0.0201	0.3068
DUS subset						
Week 12	31	105.290 (42.3711)	31	-16.419 (26.9642)	< 0.0001	0.9164
Week 24	26	95.096 (41.2235)	26	-26.538 (33.1373)	0.0001	0.0598
Control group						
Baseline	52	119.913 (35.1452)				
Week 12	52	105.548 (37.0638)	52	-14.365 (41.9712)	0.0062S	n/a
Week 24	45	107.711 (34.7214)	45	-9.844 (31.0447)	0.0295	n/a
*Within-group p-value comparing the mean score at the follow-up visit vs the mean score at baseline. †Between-group p-value comparing the mean change from baseline for the indicated test group vs the corresponding change for the control group. MITT = Modified Intent to Treat; OSS = on!® switchers subset; DUS = dual-use subset.
